# Autosomal Dominantly Inherited GREB1L Variants in Individuals with Profound Sensorineural Hearing Impairment

**DOI:** 10.3390/genes11060687

**Published:** 2020-06-23

**Authors:** Isabelle Schrauwen, Khurram Liaqat, Isabelle Schatteman, Thashi Bharadwaj, Abdul Nasir, Anushree Acharya, Wasim Ahmad, Guy Van Camp, Suzanne M. Leal

**Affiliations:** 1 Center for Statistical Genetics, Sergievsky Center, Taub Institute for Alzheimer’s Disease and the Aging Brain, and the Department of Neurology, Columbia University Medical Center, New York, NY 10032, USA; tb2890@cumc.columbia.edu (T.B.); aa4471@cumc.columbia.edu (A.A.); sml3@cumc.columbia.edu (S.M.L.); 2 Department of Biotechnology, Faculty of Biological Sciences, Quaid-i-Azam University, Islamabad 45320, Pakistan; khurramliaqat89@gmail.com; 3 Department of ENT, St-Augustinus Hospital Antwerp, 2610 Antwerp, Belgium; Isabelle.Schatteman@gza.be; 4 Synthetic Protein Engineering Lab (SPEL), Department of Molecular Science and Technology, Ajou University, Suwon 443-749, Korea; anasirqau@gmail.com; 5 Department of Biochemistry, Faculty of Biological Sciences, Quaid-i-Azam University, Islamabad 45320, Pakistan; wahmad@qau.edu.pk; 6 Center of Medical Genetics, University of Antwerp & Antwerp University Hospital, 2650 Antwerp, Belgium; guy.vancamp@uantwerpen.be

**Keywords:** autosomal dominant inheritance, exome sequencing, *GREB1L*, profound nonsyndromic hearing impairment, cochlear aplasia, cochlear nerve aplasia, neural crest, neurocristopathy

## Abstract

Congenital hearing impairment is a sensory disorder that is genetically highly heterogeneous. By performing exome sequencing in two families with congenital nonsyndromic profound sensorineural hearing loss (SNHL), we identified autosomal dominantly inherited missense variants [p.(Asn283Ser); p.(Thr116Ile)] in *GREB1L*, a neural crest regulatory molecule. The p.(Thr116Ile) variant was also associated with bilateral cochlear aplasia and cochlear nerve aplasia upon temporal bone imaging, an ultra-rare phenotype previously seen in patients with de novo *GREB1L* variants. An important role of GREB1L in normal ear development has also been demonstrated by *greb1l*^−/−^ zebrafish, which show an abnormal sensory epithelia innervation. Last, we performed a review of all disease-associated variation described in *GREB1L*, as it has also been implicated in renal, bladder and genital malformations. We show that the spectrum of features associated with *GREB1L* is broad, variable and with a high level of reduced penetrance, which is typically characteristic of neurocristopathies. So far, seven *GREB1L* variants (14%) have been associated with ear-related abnormalities. In conclusion, these results show that autosomal dominantly inherited variants in *GREB1L* cause profound SNHL. Furthermore, we provide an overview of the phenotypic spectrum associated with *GREB1L* variants and strengthen the evidence of the involvement of *GREB1L* in human hearing.

## 1. Introduction

Childhood hearing impairment (HI) is associated with impaired language acquisition, learning, speech development and affects 34 million children worldwide (World Health Organization). Approximately 1/1000 children are born with hearing loss, of which approximately 80% is genetic [1]. HI can be part of a syndrome with the presence of other medical anomalies, or it can be nonsyndromic. Currently, 120 nonsyndromic HI genes have been identified, with 59% having an autosomal recessive (AR), 37% an autosomal dominant (AD), and 5% an X-linked mode of inheritance (Hereditary hearing loss homepage). However, many genes remain to be identified due to the complexity of the hearing system and due to the understudy of some ancestries [2]. 

Nonsyndromic HI has no association with additional features or abnormalities. However, it can be associated with abnormalities of the middle ear and/or inner ear [1]. A large number of these abnormalities are mild, but bilateral cochlear aplasia, i.e., bilateral absence of the cochlea, is an ultra-rare and severe developmental abnormality of the inner ear. Approximately 0.3% of children with congenital sensorineural HI are estimated to have bilateral cochlear aplasia [3]. However, this estimate is predominately based on children who were candidates for a cochlear implant, and they usually present with severe-to-profound HI [3,4,5]. 

We previously identified de novo loss-of-function variants in *GREB1L* in two individuals with profound nonsyndromic HI with inner ear and cochleovestibular nerve (or 8th cranial) malformations (Table 1) [5,6]. Affected individuals had either absent cochleae bilaterally [p.(Glu1410fs)] or an absent cochlea on the right and incomplete partition type I on the left [p.(Arg328*)]. Both individuals also displayed abnormalities of their vestibules and absent 8th cranial nerves [6]. In addition, *greb1l*^−/−^ zebrafish exhibit a loss of and/or abnormal sensory epithelia innervation, including a loss of the anterior cristae nerve and an abnormal innervation pathway from the occipital lateral line neuromast. These findings in humans and model organisms confirm the importance of *GREB1L* in sensory innervation [6]. Furthermore, Greb1l is widely expressed during craniofacial development, including the otic vesicle [6,7], and *Greb1l*^−/−^ mice are embryological lethal and demonstrate severe abnormalities, including craniofacial and renal abnormalities [8]. *Greb1l*^+/−^ mice show an abnormal embryo size, growth retardation [9] and mild abnormalities to their kidneys and ureters [8].

In addition, de novo or autosomal dominantly inherited variants (often with reduced penetrance) have previously been implicated in individuals with renal, bladder and genital malformations [8,13,14]. Renal hypoplasia/aplasia 3 (RHDA3) is a severe developmental disorder characterized by abnormal kidney development and is caused by heterozygous *GREB1L* variants. Although the phenotype can be highly variable, the disorder falls within the most severe end of the spectrum of congenital anomalies of the kidney and urinary tract. In many of these cases, children were aborted or stillborn due to the severity of the malformations, such as bilateral renal aplasia [8,13,14]. 

In this article, we have, for the first time, identified a family with congenital profound HI that segregates a missense variant in *GREB1L* with an AD mode of inheritance and also report on an additional case with bilateral cochlear and cochlear nerve aplasia with a *GREB1L* variant.

## 2. Materials and Methods 

### 2.1. Patient Recruitment and Clinical Assessment

**Figure 1:**
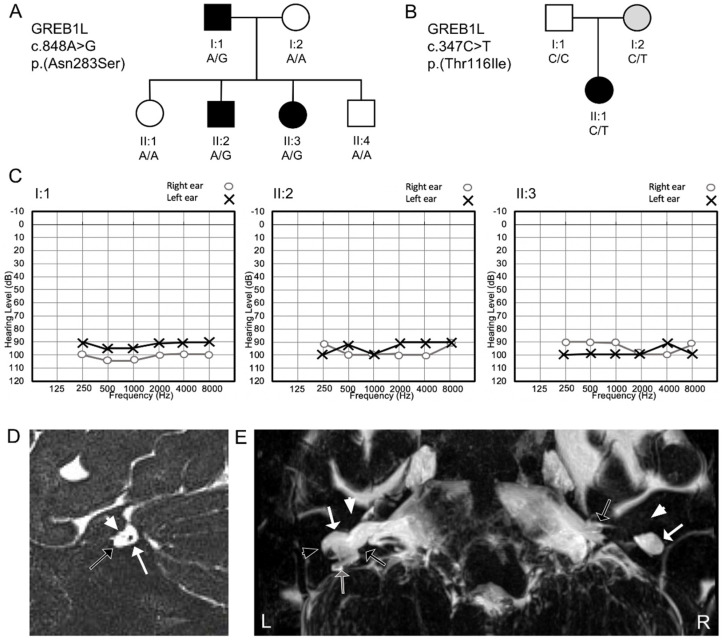
Segregation of the *GREB1L* missense variants in both families, audiological and imaging data. **A/B**. Segregation of the p.(Asn283Ser) *GREB1L* variant in family 1 (4697) (**A**) and p.(Thr116Ile) variant in family 2 (BAIE1) (**B**). Solid black symbols represent affected individuals and clear symbols unaffected family members. Grey symbols represent unaffected individuals that are also heterozygous for the variant (reduced penetrance). Females are represented by circles and males by squares. (**C**) Pure-tone audiograms of hearing-impaired family members of family 1 illustrate that each one presents with bilateral profound HI. (**D**) Oblique sagittal T2 sequence across the right internal auditory canal (IAC) of patient II:1 of family 2. The white arrow indicates the vestibular nerve (black dot). The arrowhead indicates a hypoplastic facial nerve (small grey dot). The black arrow indicates the area in the IAC where the cochlear nerve is expected but not observed. (**E**) Maximum intensity projection of a heavily T2 weighted sequence to the inner ear of the affected individual (II:1) of family 2. Bilateral cochlear aplasia and dysplasia of the vestibular system is visualized. The white arrowhead indicates the area where the cochlea is expected but not seen (bilaterally). The white arrow indicates a dysplastic cystic dilated vestibule on each side. The black arrow indicates a left narrow IAC and the right broad IAC with wide communication between the fundus of the IAC and the vestibule. The black arrowhead indicates a right dilated lateral semicircular canal. The grey arrow indicates a right rudimentary posterior semicircular canal. L, Left; R, right.

The study was approved by the ethics committee of the Quaid-i-Azam University (IRB-QAU-153), University of Antwerp (B3002020000073) and the Institutional review board of Columbia University (IRB-AAAS2343). Informed consent and peripheral blood samples were obtained from all individuals of a non-consanguineous Pakistani family with deafness (Family 1 [4697]; Figure 1A) and a non-consanguineous Egyptian family with deafness (Family 2 [BAIE1]; Figure 1B). DNA was extracted using a phenol-chloroform procedure for the Pakistani family [15] and using magnetic beads with the chemagic™ blood DNA kit on a chemagic™ Prime™ instrument (PerkinElmer, Waltham, MA, USA) for the Egyptian family. The patient evaluation included a clinical history, physical, audiological and vestibular examination. Computed tomography (CT) and magnetic resonance imaging (MRI) of the temporal bone were performed in the Egyptian patient to identify the presence of cochleovestibular malformations (Family 2). Unfortunately, we were unable to perform CT or MRI on the Pakistani family (Family 1) due to the remote location of these individuals in the Khyber Pakhtunkhwa province, Pakistan.

### 2.2. Exome Sequencing

For family 1, Sanger sequencing was performed to exclude coding variants in the HI gene *GJB2* prior to exome sequencing. Additional variants were also excluded by Sanger sequencing that are common causes of HI in the Pakistani population: i.e., p.(Phe91Ser) and p.(Cys99Trp) within *CIB2*, two intronic variants in *HGF* (c.482+1986_1988delTGA and c.482+1991_2000delGATGATGAAA) and p.(Gln446Arg) and p.(Val239Asp) in *SLC26A4* [16,17,18]. Next, a DNA sample from the affected member (II:2, family 1) underwent exome sequencing. From family 2, the affected patient (II:2) and both normal hearing parents (I:1; I:II) underwent exome sequencing. In short, exomic library preparation was performed using the SureSelect human all exon V6 kit (60.46 Mb target region) for family 1 and SeqCap EZ Exome Probes v3 (64 Mb target region) for family 2. Paired-end sequencing was performed on a HiSeq2500/4000 instrument (Illumina Inc, San Diego, CA, USA), with an average sequencing depth of on target regions of 61× for family 1 (II:2) and 119× (II:1), 97× (I:1), 106× (I:2) for family 2 and the fraction of targets covered >10× was 98.95% for family 1 and 96.80% for family 2. After removing low-quality reads, the filtered reads were aligned to the human reference genome (GRCh37/Hg19) using Burrows–Wheeler Aligner-MEM (BWAv0.7.15) [19]. Duplicate reads were marked using Picard-tools (v2.5.0). An insertions/deletion (Indel)-realignment and base quality score recalibration were performed with Genome Analysis Toolkit (GATK) (v3.7), and single nucleotide variants (SNVs) and InDels were called by the GATK HaplotypeCaller [20]. Variant annotation and filtering were performed using ANNOVAR [21]. In short for the analysis, (1) exonic and splice region variants +/− 12 bp from intron-exon boundary were retained; (2) An AD mode (including de novo for family 2) and AR mode of inheritance was considered for both families; (3) Variants with a predicted effect on protein function or pre-mRNA splicing (missense, nonsense, frameshift, start-loss, splice region, etc.) with a population-specific minor allele frequency (MAF) of <0.005 (for AR) and <0.0005 (for AD) in all populations of the Genome Aggregation Database (gnomAD) [22] and the Greater Middle East Variome Project (GME) [23] were retained to test for segregation; and (4) Bioinformatic prediction scores were annotated from dbnsfp35a and dbscSNV1.1 to evaluate missense and splice site variants respectively [24,25], including Combined Annotation Dependent Depletion (CADD) and Genomic Evolutionary Rate Profiling (GERP++) scores [26,27]. Genes previously involved in human/animal HI or genes expressed in the inner ear were prioritized [28,29]. Candidate variants obtained from filtering were visualized with the Integrative Genomics Viewer (IGV2.4.3). Sanger sequencing performed using an ABI3130XL Genetic Analyzer was used to validate the variants in both families and check the segregation of variants in additional family 1 members for which DNA was available. 

Copy number variants (CNVs) were called in exome data from both families using CONiFER (v0.2.2) [30]. Gene annotation was done using the BioMart Database [31] and variant frequency was assessed using the Database of Genomic Variants [32] and gnomAD [22] using the same frequency cut-offs as above for SNV/InDels. 

## 3. Results

### 3.1. Clinical Findings

In the Pakistani family (Family 1), hearing impairment was prelingual for the three affected family members, and pure-tone audiometry revealed bilateral profound sensorineural HI (Figure 1). No gross vestibular dysfunction was observed via a tandem gait test, and Romberg test in affected individuals I:1, II:2, and II:3. Clinical histories were obtained, and the patients underwent a physical exam at the ages of 45 years of age (y) (I:2), 15y (II:2), and 17y (II:3) with no other health problems reported, including no kidney or bladder issues, however asymptomatic kidney disease could not be excluded. History of head trauma, severe infections or ototoxic treatment was not present. None of the other family members displayed HI or any other clinical features, and the parents have no reported consanguinity.

In the Egyptian patient (Family 2), auditory brainstem responses and cochlear microphonic potentials were bilaterally absent. Vestibular testing showed bilateral aberrant head impulse test, minimal nystagmi on the rotational chair test, and no nystagmus response on the caloric test (with water 44 °C), suggesting a reduced canalar function. C-Vemp (cervical-vestibular evoked myogenic potentials) were bilaterally present at 130 dBSPL, implying a functioning vestibule. MRI and CT imaging showed bilateral cochlear aplasia, aplasia of the cochlear nerve and dysplasia of the vestibule and semicircular canals (Figure 1). The vestibular nerve was present on both sides. On the left side, a hypoplastic narrow internal auditory canal (IAC) was found. On the right side, the IAC was wide with deficient fundus and wide communication between the IAC and vestibule. The facial nerve had a hypoplastic aspect bilaterally. The patient did not have any other known health issues, however, mild kidney disease could not be excluded. The parents have normal hearing, reported no health issues and are non-consanguineous. There is no family history of congenital or progressive HI.

### 3.2. Exome Sequencing

In family 1, exome sequencing and variant filtering identified variants in *MYO15A*, *POLE* and *GREB1L* as candidates and were validated and tested for segregation (Table S1). Only a missense [(NM_001142966.2:c.848A>G:p.(Asn283Ser)] variant in *GREB1L*, a gene previously associated with HI, segregated with HI in pedigree 4697 with an AD mode of inheritance (Table 1, Figure 1, Figure S1) [6]. The variant is absent from gnomAD and GME. It is located at a conserved position amongst species (GERP++ RS: 3.44; phastCons20way_mammalian: 1.00). The variant has a CADD score = 10 and is predicted damaging by fathmm-MKL. Based on ESEfinder (v2.0) [10], the variant is located in an exonic splicing enhancer motif (ACAGTAG; score 2.74; threshold >2.67) predicted responsive to Pre-MRNA-Splicing Factor SRp40, which is lost due to the variant (GCAGTAG; score 1.28; threshold >2.67). Therefore, the variant might impact normal protein functioning through various mechanisms, however, we do not know its exact effect in vivo as we were unable to obtain RNA from the patients. *GREB1L* is intolerant to loss-of-function (LoF) variants and is likely under selection against them (pLI = 1; o/e = 0.02 [0.01–0.07]) [22], with only 2% of the expected LoF variants observed. In addition, only 52% of the expected missense variants are observed in *GREB1L* (z score = 5.37; o/e = 0.52 [0.49–0.56]) [22]. The p.(Asn283Ser) missense variant is located in a position and region with a gene-specific missense tolerance ratio (MTR) percentile of <25 [33], which signifies that this region of the protein is also less likely to tolerate missense variants. Pathogenic missense variants are enriched within the 25th percentile of the intolerant region of the gene’s MTR distribution [33]. 

In family 2, all family members were sequenced via exome sequencing. We also identified a variant in *GREB1L* [(NM_001142966.2:c.347C>T:p.(Thr116Ile)], which was verified with Sanger sequencing (Figure S1). None of the other identified variants were likely to be related to HI (Table S1). The p.(Thr116Ile) variant in *GREB1L* was inherited from the unaffected mother, and the variant is absent from gnomAD and GME. It is located at a conserved position amongst species (GERP++ RS: 5.25; phastCons20way_mammalian: 0.935), has a CADD score = 30, and is predicted damaging by fathmm-MKL. The temporal bone imaging phenotype of the patient in this family is remarkably similar to patients previously described with *de novo GREB1L* variants (Table 1), a phenotype that is ultra-rare [6]. 

Based on the ACMG guidelines for variant classification, p.(Asn283Ser) was classified as likely pathogenic (PM2, PP1-M [Bayes Factor = 16 [34]], PP2 and PP3) and p.(Thr116Ile) was classified as a variant of unknown significance (PM2, PP2, PP3 and PP4) [12]. Finally, no CNVs were identified in either family that were likely to be involved in disease etiology.

### 3.3. Phenotypic Spectrum of GREB1L Variation

**Figure 2:**
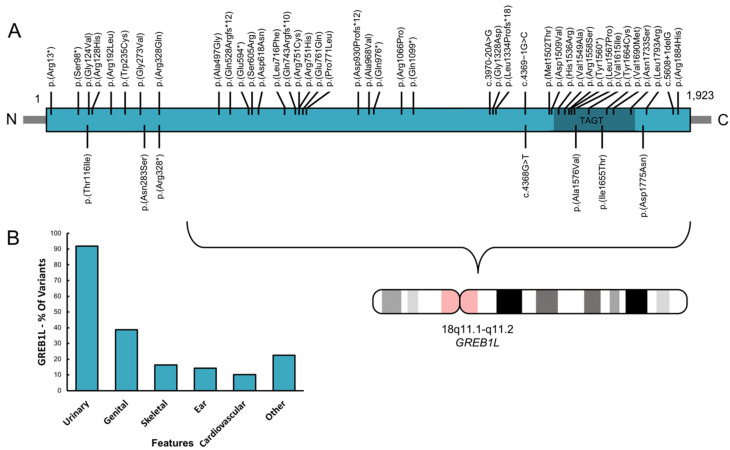
All variants reported in GREB1L and their associated phenotypic features. **A**. GREB1L protein structure with all variants indicated. The seven bottom variants are associated with ear-related abnormalities. Dark green, TAGT or Ten-eleven translocation/J binding protein (TET/JBP)-associated glycosyltrasferase domain [39]. **B**. The percentage (%) of variants associated with the most prevalent phenotypic features seen in affected individuals.

We performed a detailed literature search of all disease-associated variation (N = 49) reported in *GREB1L*, which are listed in Table 2 and displayed in Figure 2. This illustrates that variants are present over the entire length of the gene, with some clustered in or near the TAGT domain. Although previous studies were mostly focused on renal malformations, this table shows that a variety of malformations can be present in affected individuals, including renal, bladder, uterus, ear and other issues such as skeletal abnormalities. Reduced penetrance was observed in 50% of the reported variants in which parents or unaffected siblings were also assessed. In addition, variants were inherited maternally in a large majority of cases (71%). Three patients previously studied for renal malformations also showed ear-related issues. Therefore, of the total of 49 variants that have been reported, 7 variants (14%) have been associated with a hearing or an ear abnormality.

## 4. Discussion

HI in children is both genetically and phenotypically heterogeneous. Identification of novel genes implicated in congenital HI is important to understand normal hearing and ear development, for patient management and intervention and for the development of novel therapeutic strategies.

We identified two families with congenital profound nonsyndromic sensorineural HI that segregate missense variants [p.(Asn283Ser) and p.(Thr116Ile)] in *GREB1L* (Figure 1). GREB1L is a premigratory neural crest (NC) regulatory molecule implicated in the embryonic development of many tissues [40]. The cranial NC is important in the development of the peripheral nervous system and non-neural tissues, including craniofacial connective and skeletal tissues [41]. In addition, it also gives rise to the stria vascularis of the inner ear and the glia cells of the cochleovestibular nerve and inner ear ganglion [42]. *greb1l* has also been implicated in Hoxb1 and Shh_a_ signaling in zebrafish [14], important pathways in the inner ear and cranial nerve development [43,44,45].

Previous reports on disease-related *GREB1L* variants showed that a variable phenotype is present, including within families segregating the same variant (e.g., left vs. right ear) [36,37]. In addition, a high level of reduced penetrance has been reported, including in family 2 of this study. There is no evidence that variants cluster within specific domains of the protein (Table 2; Figure 2). This finding is similar to what was observed for *EYA1*, an NC regulatory molecule which is involved branchio-oto-renal (BOR)/branchio-otic (BO) syndrome etiology [46]. *EYA1* is also characterized by a high level of phenotypic variation between patients, even within the same family, and the severity of the phenotype does not correlate with the type of variant nor with the domain involved. In BOR patients with *EYA1* variants, which presents with both ear and renal abnormalities, normal kidneys were often observed in family members with BOR while other family members had renal abnormalities [46]. Many neurocristopathies typically show this variable phenotypic profile amongst patients, even within families or within the same individual (left vs. right) [47], and multiple hypotheses have been suggested to explain this phenomenon, such as environmental factors and genetic modifiers [6,46,48]. However, as the NC is a transient and migratory cell population during development, there are also complex micro-regulations that could disturb NC migration during development. Because of this, the path of NC migration that ends up affected due to *GREB1L* dysfunction could perhaps be attributed to chance. An example of this can be found in knockout (Wv/Wv) mice. These mice have a defect in c-kit, a NC migration regulatory molecule involved in the migration and proliferation of melanocytes in the inner ear. Wv/Wv mice show uni- or bilateral inner ear issues with variable hearing levels, and this variability in inner-ear phenotype was found to be reflected by the number of melanocytes present and how far they migrated along each cochlea during development [49]. 

The particular link between renal and ear abnormalities has previously been demonstrated [47], including in neurocristopathies. Several neural crest regulatory molecules are known to cause ear/kidney syndromes with variable expression of both ear and kidney phenotypes (e.g., *EYA1*, *SIX1*, *SIX5*, *CHD7*, *MASP1*, *TBX1*), involved in BOR/BO syndrome, CHARGE syndrome, 3MC syndrome and DiGeorge syndrome [5,47]. In addition to these, there are also several other disorders with a specific renal/ear link, such as Alport syndrome and Bartter syndrome [50,51]. Interestingly, when reviewing all variants reported in *GREB1L* to date, we also demonstrate that 14% of *GREB1L* variants (N = 7) have been associated with ear-related issues. It is to be noted however, that many of the previous reports (focused on renal malformations) included aborted/stillborn fetuses, in which hearing could not have been assessed. In addition, inner ear and cochlear nerve malformations cannot be assessed via prenatal ultrasound and if an autopsy was performed and would usually not be detected on routine autopsy. Therefore, the number of ear malformations associated with *GREB1L* variants is likely under-reported. Last, this renal/ear link is also seen in Mayer-Rokitansky-Kuster-Hauser (MRKH) syndrome, characterized by abnormal development of the internal reproductive system in females, and is also caused by *GREB1L* variants (Table 2). Interestingly, HI is reported in 10–25% of individuals with MRKH syndrome [37]. 

We also detected a maternal bias in the inheritance of *GREB1L* variants (Table 2). This maternal bias has previously observed and two mechanisms have been suggested: (1) imprinting [8,36] (2) or *GREB1L* variants could affect male fertility resulting in a low rate of paternal inheritance [8]. Genital issues, including uterus aplasia, are common and have been reported in many females (Table 2), but the presence in males may be underestimated as the defect might not be a gross morphological abnormality that causes infertility.

De novo *GREB1L* variants have been previously implicated in a phenotype which consists of profound HI and inner ear and cochleovestibular nerve malformations [6]. The inner ear malformation seen in family 2 is remarkably similar to the patients previously reported with *de novo GREB1L* variants (Table 1) [6], and includes cochlear aplasia, cochlear nerve aplasia and bilateral dysplastic vestibules and semicircular canals (Figure 1), an ultra-rare phenotype. The finding of multiple independent cases with *GREB1L* variants and this exact ultra-rare phenotype is significant [6]. In addition, *greb1l*^−/−^ zebrafish (p.Gln408Ter) exhibit a loss of or abnormal sensory epithelia innervation [6], supporting the importance of *GREB1L* in the inner ear and nerve development.

Unfortunately, we were unable to perform temporal bone imaging in the affected members of family 1 since they are located in a remote village in Pakistan. The profound bilateral congenital HI phenotype observed for affected members of this family suggests that it may also be due to inner ear/cochleovestibular nerve malformations. Since sample collection for DNA extraction and genetic screening is easier to implement in areas with limited access to modern healthcare systems than temporal bone imaging, we believe including *GREB1L* in diagnostic screening for nonsyndromic HI is valuable.

In conclusion, we demonstrate that autosomal dominantly inherited variants in *GREB1L* are involved in profound sensorineural HI etiology and show that *GREB1L* behaves with a similar phenotypic variance compared to other neurocristopathies. In addition, we recommend including *GREB1L* in diagnostic screening panels for nonsyndromic HI.

## Figures and Tables

**Table 1:** Variants identified in *GREB1L* associated with nonsyndromic hearing impairment.

Family Type	Variant Segregation	Inheritance Model	Predicted Variant Effect	cDNA Change ^1^	AA Change	gnomAD	CADD Score (v1.3)	GERP++RS	Splicing Effect Prediction ^2^	Phenotype	ACMG ^3^	Study
**Trio**	de novo	AD	splicing	c.4368G>T	p.(Glu1410fs)	absent	26	5.17	splice site loss	profound bilateral SNHI; UCA (right); UIP-I (left); BVES + SCC; BCNA	P	[6]
**Trio**	de novo	AD	nonsense	c.982C>T	p.(Arg328*)	absent	38	4.52	NA	profound bilateral SNHI; BCA; BVES + SCC; BCNA	P	[6]
**Family**	inherited	AD	missense/splicing	c.848A>G	p.(Asn283Ser)	absent	10	3.44	ESE site loss	profound bilateral SNHI ^4^	LP	This study (Family 1)
**Trio**	inherited ^5^	AD	missense	c.347C>T	p.(Thr116Ile)	absent	30	5.25	NA	profound bilateral SNHI; BCA; BVES + SCC; BCNA	VUS	This study (Family 2)

^1^ Based on NM_001142966.2; ^2^ Based on Human splicing finder (v.3.1), ESEfinder (v2.0) [10,11]. ^3^ Classified based on the American College of Medical Genetics (ACMG) guidelines: P, Pathogenic; LP, likely pathogenic; VUS, variant of unknown significance [12]. ^4^ Inner ear not evaluated via imaging. ^5^ Maternal reduced penetrance; AD, Autosomal Dominant; BCA, bilateral cochlear aplasia; BCNA, bilateral cochlear nerve aplasia; BVES + SCC: bilateral dysplastic vestibule and semicircular canals; ESE, exonic splicing enhancer; NA, Not applicable; SNHI, sensorineural hearing impairment; UCA, unilateral cochlear aplasia; UIP-I, unilateral incomplete partition type I.

**Table 2:** All variants reported in *GREB1L* and their associated phenotypic features.

cDNA Variant ^1^	Amino Acid Variant	Urinary Phenotype	Genital Phenotype	Ear Phenotype ^2^	Other Phenotypes	Inheritance	Reduced Penetrance	Reference
c.37C>T	p.(Arg13*)	unilateral MCD, congenital megaureter	–	–	–	NA	NA	[14]
c.293C>G	p.(Ser98*)	BKA	–	–	–	de novo	no	[13]
c.347C>T	p.(Thr116Ile)	–	–	profound bilateral SNHI, BCA; BVES+SCC; BCNA	–	mat	yes (mat)	This study
c.371G>T	p.(Gly124Val)	BKA, UKA, bladder hypoplasia	–	–	Potter sequence	suspected pat ^4^	NA	[35]
c.383G>A	p.(Arg128His)	UKA	unicornate uterus, agenesis of left ovary	–	–	NA	NA	[14]
c.575G>T	p.(Arg192Leu)	BKA, UKA	unique fallopian trump and ovary	–	insulin-dependent diabetes	mat	no	[8]
c.705G>T	p.(Trp235Cys)	BKA, UKA, renal cysts, clear cell renal carcinoma	MRKH, arcuate uterus	–	–	AD family (2 mat, 2 pat)	yes (2 mat, 1 unaffected female sib)	[36]
c.818G>T	p.(Gly273Val)	UKA	–	–	–	NA	NA	[14]
c.848A>G	p.(Asn283Ser) + splicing	–	–	profound bilateral SNHI ^3^	–	AD family (1 pat)	no	This study
c.982C>T	p.(Arg328*)	–	–	profound bilateral SNHI, BCA; BVES+SCC; BCNA	–	de novo	no	[6]
c.983G>A	p.(Arg328Gln)	pelvic kidney, MCD, VUR		–	–	mat	no	[8]
c.1490C>G	p.(Ala497Gly)	UKA	–	–	–	NA	NA	[14]
c.1582delC	p.(Gln528Argfs*12)	BKA, UKA	UA, unicornuated uterus	–	clinodactyly	mat	yes (mat)	[8]
c.1780G>T	p.(Glu594*)	BKA, UKA, VUR	UA, fallopian trumps absence, ovarian hernia, uterine left artery absent	–	–	mat	no	[8]
c.1813A>C	p.(Ser605Arg)	UKA, multilocular cyst	blind ending hemi-vagina and bicornuated uterus	–	–	pat	yes (pat)	[8]
c.1852G>A	p.(Asp618Asn)	Ectopic kidney, VUR, duplicated ureter	MRKH type 2	–	unilateral polydactyly, facial asymmetry	NA	NA	[37]
c.2148G>T	p.(Leu716Phe)	VUR	–	–	iris anomaly	NA	NA	[8]
c.2227del	p.(Gln743Argfs*10)	UKA, MCD	MRKH type 2, UA	–	scoliosis	AD family (1 mat)	yes (mat)	[37]
c.2251C>T	p.(Arg751Cys)	BKA, unilateral hypoplasia	unicornuated uterus	–	–	mat	no	[8]
c.2252G>A	p.(Arg751His)	UKA, MCD, megaurethra	–	–	hepatic portal fibrosis	mat ^5^	NA	[8]
c.2281G>C	p.(Glu761Gln)	UKA, duplication of the ureter, unilateral MCD, congenital megaureter	–	–	–	pat	yes (pat)	[14]
c.2312C>T	p.(Pro771Leu)	UKA, MCD	UA, streak ovaries, rudimentary follopian tubes	–	Right unique umbilical artery, 11 pairs of ribs, 6 cervical hemivertebrae with 1 hemivertebrae	NA	NA	[37]
c.2787_2788del	p.(Asp930Profs*12)	BKA, UKA, MCD	UA	–	–	AD family (1 mat; 1pat)	yes (mat)	[37]
c.2903C>T	p.(Ala968Val)	BKA, with agenesis of ureters, bladder hypoplasia	–	–	–	de novo	no	[13]
c.2926C>T	p.(Gln976*)	BKA, UKA	–	–	–	mat	no	[8]
c.3197G>C	p.(Arg1066Pro)	UKA	–	–	–	NA	NA	[14]
c.3295C>T	p.(Gln1099*)	unilateral MCD	–	–	–	mat	no	[14]
c.3970-20A>G	splicing	BKA, ureter and bladder aplasia	UA	–	Unilateral hexadactyly	pat	yes (pat)	[37]
c.3983G>A	p.(Gly1328Asp)	UKA	MRKH type 2	–	–	NA	NA	[37]
c.3998_3999insC	p.(Leu1334Profs*18)	UKA	–	–	–	mat	no	[14]
c.4368G>T	splicing	–	–	profound bilateral SNHI, UCA (right); UIP-I (left); BVES+SCC; BCNA	–	de novo	no	[37]
c.4369−1G>C	splicing	BKA	UA	–	thickened left ventricular wall, 10 pairs of ribs	mat	yes (mat)	[8]
c.4505T>C	p.(Met1502Thr)	BKA	–	–	retro-esophageal subclavian artery, adrenal gland hypoplasia, enlarged thymus, one pair of cervical ribs	mat ^5^	NA	[8]
c.4526A>T	p.(Asp1509Val)	BKA	–	–	adrenal cytomegaly	NA	NA	[8]
c.4607A>G	p.(His1536Arg)	BKA, UKA, MCD, horseshoe kidney	UA	–	11 pairs of ribs	mat	no	[8]
c.4646T>C	p.(Val1549Ala)	UKA	UA	–	Henoch Schönlein Purpura	NA	NA	[14]
c.4672C>A	p.(Arg1558Ser)	BKA	–	–	–	mat	yes (mat)	[8]
c.4680C>A	p.(Tyr1560*)	BKA, bladder agenesis, VUR	uterus anomaly	–	Potter sequence	AD family (1 mat)	no	[14]
c.4700T>C	p.(Leu1567Pro)	UKA	–	–	–	de novo	no	[14]
c.4727C>T	p.(Ala1576Val)	BKA	–	auricular tag	hypertrophic left ventricle, aortic stenosis	NA	NA	[8]
c.4843G>A	p.(Val1615Ile)	BRHD, congenital hydronephrosis	–	–	–	mat	yes (mat)	[14]
c.4964T>C	p.(Ile1655Thr)	UKA	–	unilateral SNHI	genu valgum, flat feet	pat	yes (pat)	[14]
c.4991A>C	p.(Tyr1664Cys)	UKA	–	–	–	NA	NA	[14]
c.5068G>A	p.(Val1690Met)	UKA, VUR	–	–	–	pat ^5^	NA	[14]
c.5198A>G	p.(Asn1733Ser)	BKA, UKA, ureter and bladder aplasia	UA, hemi-uterus, streak ovaries	–	–	AD family (1 mat, 1 pat)	yes (mat; unaffected female sib)	[37]
c.5323G>A	p.(Asp1775Asn)	BKA	–	preauricular tag, lop ear	–	NA	NA	[8]
c.5378T>G	p.(Leu1793Arg)	BKA, RKA, hypertrophy of the kidney	–	–	–	AD family (2 mat)	yes (1 mat)	[38]
c.5608+1delG	splicing	BKA, UKA, bladder agenesis	undifferentiated external female genitalia	–	Potter sequence	de novo, mat	yes (2 male siblings)	[35,38]
c.5651G>A	p.(Arg1884His)	URHD	–	–	–	NA	NA	[14]

^1^ Based on NM_001142966.2. ^2^ Many of the children with renal malformations listed here were aborted/stillborn, in which hearing could not have been assessed. ^3^ Inner ear not evaluated via imaging. ^4^ No genetic evaluation. ^5^ Parent not evaluated via renal ultrasound. AD, Autosomal Dominant; AD family, family with multiple affected (>2) showing autosomal dominant inheritance; BCA, bilateral cochlear aplasia; BCNA, bilateral cochlear nerve aplasia; BKA, bilateral kidney agenesis; BVES + SCC, bilateral dysplastic vestibule and semicircular canals; ESE, exonic splicing enhancer; mat, maternal inheritance; MCD, multi-cystic dysplasia; MRKH, Mayer-Rokitansky-Küster-Hauser syndrome; NA, Not assessed; pat, paternal inheritance; SNHI, sensorineural hearing impairment; UCA, unilateral cochlear aplasia; UIP-I, unilateral incomplete partition type I; UKA, unilateral kidney agenesis; UA, uterovaginal aplasia or uterus aplasia; VUR, vesicoureteral reflux.

## Supplementary Materials

The following are available online at https://www.mdpi.com/2073-4425/11/6/687/s1, Table S1: Candidate variants identified in both families, genotypes and annotations. Figure S1: Sanger traces of the *GREB1L* variants in both families.

## Web Resources

ANNOVAR	http://annovar.openbioinformatics.org/
Burrows-Wheeler aligner	http://bio-bwa.sourceforge.net/
ClinVar	https://www.ncbi.nlm.nih.gov/clinvar/
combined annotation dependent depletion (CADD)	http://cadd.gs.washington.edu/
dbSNFP	https://sites.google.com/site/jpopgen/dbNSFP
dbSNP	https://www.ncbi.nlm.nih.gov/projects/SNP/
gEAR	https://umgear.org/
genome aggregation database (gnomAD)	http://gnomad.broadinstitute.org/
genome analysis toolkit (GATK)	https://software.broadinstitute.org/gatk/
genomic evolutionary rate profiling (GERP)	http://mendel.stanford.edu/SidowLab/downloads/gerp/
Greater Middle East (GME) variome project	http://igm.ucsd.edu/gme
Hereditary hearing loss homepage	https://hereditaryhearingloss.org
Online Mendelian inheritance of man (OMIM)	https://www.omim.org/
PhastCons and PhyloP	http://compgen.cshl.edu/phast/
Picard	http://broadinstitute.github.io/picard/
SHIELD	https://shield.hms.harvard.edu/
World Health Organization	https://www.who.int/news-room/fact-sheets/detail/deafness-and-hearing-loss
