# Supplementary figures and images for: Autosomal Dominantly Inherited GREB1L Variants in Individuals with Profound Sensorineural Hearing Impairment
Source: Genes (Basel). 2020 Jun 23;11(6):687. doi: 10.3390/genes11060687 (PMC7349314; doi:10.3390/genes11060687)

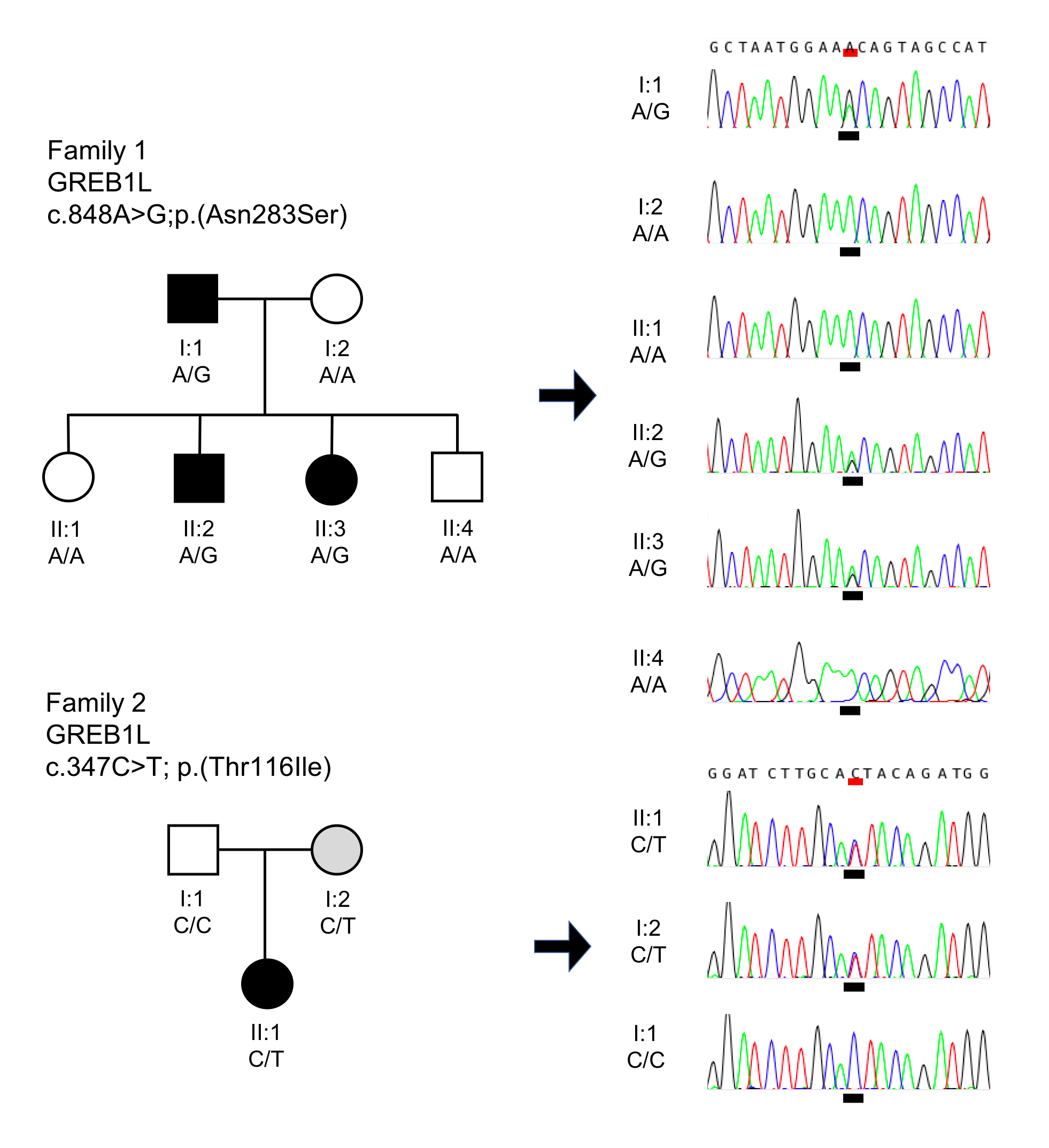

Supplement: Supplementary file 1 [file genes-11-00687-s001.zip › Figure S1.png]
